# Amorphous Carbon-Induced Surface Defect Repair for Reinforcing the Mechanical Properties of Carbon Fiber

**DOI:** 10.3390/ma12081244

**Published:** 2019-04-16

**Authors:** Dachao Li, Hongzhong Liu, Bangdao Chen, Dong Niu, Biao Lei, Guoyong Ye, Weitao Jiang, Yongsheng Shi, Lei Yin, Guoquan Lai

**Affiliations:** State Key Laboratory for Manufacturing Systems Engineering, Xi’an Jiaotong University, Xi’an 710049, China; dachaozaiwf@163.com (D.L.); dongniu@mail.xjtu.edu.cn (D.N.); biaolei@mail.xjtu.edu.cn (B.L.); guoyongye@mail.xjtu.edu.cn (G.Y.); wtjiang@mail.xjtu.edu.cn (W.J.); shiyongsheng@mail.xjtu.edu.cn (Y.S.); leiyin@mail.xjtu.edu.cn (L.Y.); wrfd92@163.com (G.L.)

**Keywords:** carbon fiber, amorphous carbon, graphene oxide, tensile strength, interfacial properties

## Abstract

Graphene oxide (GO) was prepared using metal-catalyzed crystallization of amorphous carbon on a carbon fiber surface to improve the mechanical properties of the carbon fiber (CF). The deposited GO was used for repairing of surface structure defects on CF, thereby improving the tensile strength and interfacial strength force of CF. The grown morphology of GO and the changes in CF surface microstructure before and after remediation were investigated in detail by scanning tunneling microscopy and Raman spectroscopy. The effects of surface repair on the mechanical properties of the CF and the resulting composites were investigated systematically. The results of scanning tunneling microscopy show that the graphene oxide formed on the surface of carbon fiber present uniform dispersion. Raman spectroscopy curves indicate that CF successfully remediated the defects in the CF surface. The results of mechanical properties testing show that such a remediation method could significantly enhance the tensile strength of CF and increase the interfacial strength versus raw fibers; that is, the tensile strength of CF was enhanced by 42% and the interfacial strength by 33.7%.

## 1. Introduction

Carbon fiber is an attractive type of reinforcement because of its excellent tensile strength, high stiffness, and lightness [1,2]. The demand for high-performance carbon fiber composites in the sports, automotive, aerospace, and other industries has been increasing [3,4]. Although CFs have a superior tensile strength, their practical mechanical properties—especially the tensile strength of CF—are far lower than the theoretical value. To improve the tensile strength of CF, it is necessary to study the relationship between the surface microstructure and macromechanical properties. 

Johnson states that the main factor restricting fiber tensile strength is surface defects, which is also the location of tensile fractures of fiber [5,6]. To overcome the limitations imposed by surface structural defects in CF, many researchers have developed technologies concerning surface modification [7,8]. GO is an ideal nanoscale reinforcement with outstanding mechanical, electrical, and thermal properties [9,10]. Recently, GO was shown to be a reinforcing phase in CF because it can increase CF effective cross-sectional areas and enhance mechanical interlocking between fibers and graphene [11,12]. Meanwhile, the introduction of GO increased the roughness and chemically active state. This can overcome the poor matrix compatibility and weaken the adhesion of the CF fiber matrix. 

Methods to introduce GO on the defects of CFs include coating [13], chemical grafting [14], chemical vapor deposition [15], supercritical techniques [16], and solvothermal treatment [17]. However, in the traditional process, precise control over the mass and the number of GO is difficult due to its sensitivity to various process parameters. Therefore, a method to effectively enhance the tensile strength and improve interfacial strength of CF via GO grafting is urgently needed.

Here, we report a simple and efficient method to synthesize GO and improve defects via metal-catalyzed crystallization of amorphous carbon by thermal annealing. The quality of graphene is mainly influenced by the initial thickness of the amorphous carbon layer and the thickness of catalyst film, which is in contrast to prior work. The results of scanning tunneling microscopy show that the GO formed on the surface of carbon fiber presented uniform dispersion. Raman spectroscopy curves indicate that CF successfully remediated the defects in the CF surface. The mechanical properties test results show that this method could significantly enhance the tensile strength of CF and increase the interfacial strength. Compared with raw fibers, the interfacial strength of CF showed enhancement of 33.7%, while the tensile strength increased by 42%.

## 2. The Defect Theory of CF

CF is one type of graphite; however, its actual tensile strength is much higher than the theoretical value of graphite. Based on the inherent strength theory of solid materials, Gilman derived a theory for the inherent strength of brittle materials [18], namely:

σc=0.52(Eγy0)1/2,

where $y_{0}$ is the atomic spacing and γ is the surface energy. When they are $1.42\;\overset{\cdot }{A}$ and $9.7\times 10^{-4}\;J/{\mathrm{cm}}^{2}$, respectively, the theoretical tensile strength of graphite is 180 GPa. Although existing CF products are strong, the actual strength is far from the theoretical strength because almost all solid matter has a degree of structural imperfection due to defects. For brittle materials, fracture usually occurs at these defects [19]. Defects are important factors that cause the material’s strength to decline. CF is a nearly brittle material and has almost no plastic flow characteristics. When this is inflicted on the CF tensile load, the concentrated stress is easily generated at the tip of the crack. The stress will act on the new surface in the form of crack propagation or extension until the stress is fully released. During the process of stress release, the relationship between concentrated stress and the ability of the material to withstand stress can be expressed as:

σm=[1+2(βξ)12]σ.

The concentrated stress $\sigma_{m}$ is 100-fold larger than the stress σ value because the volume of the crack itself β is several times larger than the radius of curvature of the crack tip ξ. Therefore, the crack is a potential source of fracture and one of the main factors restricting the strength.

Griffith’s theory is based on the fact that material fracture begins with a crack, and the energy released by crack propagation is equal to the energy required to form a new surface. Correlation between crack size and crack tensile strength can be expressed in the following formula:

(1) σt=[2Eγ(πβ)]12,

where $\sigma_{t}$ is the tensile strength, and β is the crack size. Formula (1) shows that the tensile strength decreases to the square power as the crack increases, i.e., a very small crack may cause CF failure. Therefore, it is necessary to repair the defects of CF to improve its performance.

## 3. Experimental

### 3.1. The Defect Repair Process of CF

**Figure 1:**
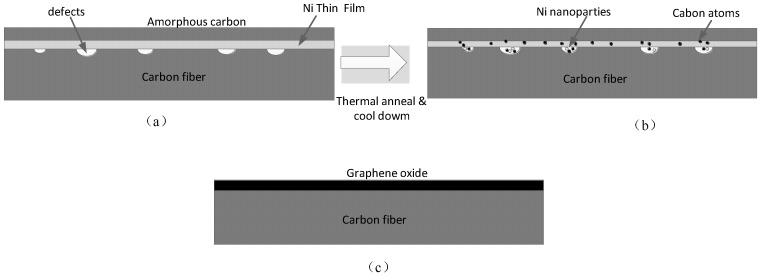
Schematics of the defect repair process of carbon fiber (CF). (**a**) Schematic of nickel and amorphous carbon deposited on defective fiber; (**b**) Graphene oxide (GO) forming process during thermal treatment; (**c**) schematic of CF after thermal anneal.

Surface structure defects of poly (acrylonitrile)-based CF were repaired with graphene (CFs purchased from Zhongfu Shenying Carbon Fiber Co., Ltd., Lianyungang, China). First, the CFs were washed with ultrasonic dewatering for 20 min and then dried for 2 h at 80 °C. The sizing agents on the CF surface were removed via thermal treatment under Ar gas. Next, see Figure 1a, nickel was used as a catalyst to sputter nickel on the surface of the desizing CF with metal sputtering equipment; the thickness of the nickel layer was controlled at 20 nm. A 30-nm amorphous carbon layer was deposited on the nickel film as an auxiliary carbon source via electron beam evaporation. Then, the CF was placed in an argon gas tube furnace and heated from 700 °C to 750 °C for 5 min with hydrogen; part of the film was converted into nanoparticles. During this process, the CF was subjected to 20 N tension. The aim of the tension is to induce nanoparticles to diffuse into the defects on the CF surface and enhance the interaction force between GO and CF, as shown Figure 1b. Hydrogen (as reducing gas) and amorphous carbon (as a carbon source) were provided to generate GO on the CF surface defects for 30 min at a furnace temperature of 750 °C. The feeding rates of argon and hydrogen were 100 and 40 sccm, respectively. Finally, the temperature was reduced to room temperature at a rate of 20 °C/s. The schematic of CF after annealing is shown in Figure 1c. The mechanism is that carbon atoms are dispersed in the fiber layer through the metal layer at a high temperature and then precipitated to a free surface (to reach a solid solution limit) during cooling. For a given annealing condition and cooling rate, the quality of GO can be easily controlled by the thickness of the initial deposited carbon layer and the amount of metal catalyst.

### 3.2. Microstructural Analysis of CF Surface

The morphology of the CF surfaces was investigated before and after annealing of the catalyst-deposited CFs using a field emission scanning electron microscopy (SEM, 200FEG, Quanta FEI Inc., Hillsboro, OR, USA). Prior to characterization, excess metallic nickel and indeterminate toner were cleaned by acid and subsequently cleaned in deionized water with an ultrasonic vibration for 10 min. The samples were then dried at 80 °C for 1 h. Raman spectra were recorded on a LabRAM ARAMIS 2208823NE (LabRAM ARAMIS, Reims, France) microscopic Raman spectrometer with a 633 nm laser. It was used to determine the quality of GO synthesized.

### 3.3. Single Fiber Tensile Test of CFs

The carbon monofilament was subjected to a tensile test according to the ASTM D3822-07 specification to determine its axial tensile strength, ultimate strength, and strain at failure.

**Figure 2:**
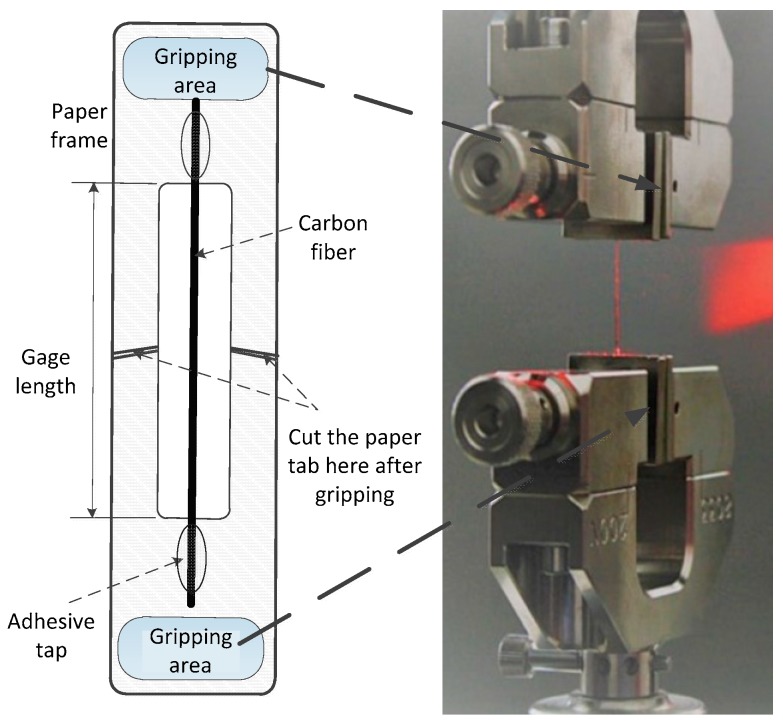
Single fiber specimen for axial tensile testing.

As shown in Figure 2, a single fiber was randomly separated from the CF bundles, and its ends were attached to paper tabs at a distance of 30 mm using an adhesive. The tab for mounting the specimen in the machine was prepared from a thick paper. A slot of length equal to 20 mm is cut out in the middle of the tab. More than 30 single fibers were tested to obtain averaged quantities. The actual gauge length of the fiber is measured using a Vernier caliper with an accuracy of less than 0.1 mm. The fiber axis was aligned with the crosshead axis to simulate the uniform stress state across the fiber cross-section.

### 3.4. Interfacial Shear Strength Test of the CFs

We next used a microdroplet to determine the interfacial shear strength (IFSS) between epoxy and CFs measured from microdroplet specimens adhered onto a single CF. We utilized CF with a diameter of 7 μm as a single fiber to make microdroplet bonded specimens. Bisphenol-A type epoxy and piperidine hardener were mixed for the matrix resin. The mixing ratio of the epoxy resin to the curing agent was 17:1, and when cured to a suitable viscosity, a sample of the single fiber microdroplet bonding experiment was prepared. The IFSS was calculated according to the below equation [20,21]:

$$IFSS=\frac{F}{\pi dl},$$

where F is the maximum load recorded, d is the CF diameter, and l is the embedded length. The microdroplet resins were cured to form a microdroplet; the single CF was fixed on paper to measure the embedded length of each microdroplet via optical microscopy.

**Figure 3:**
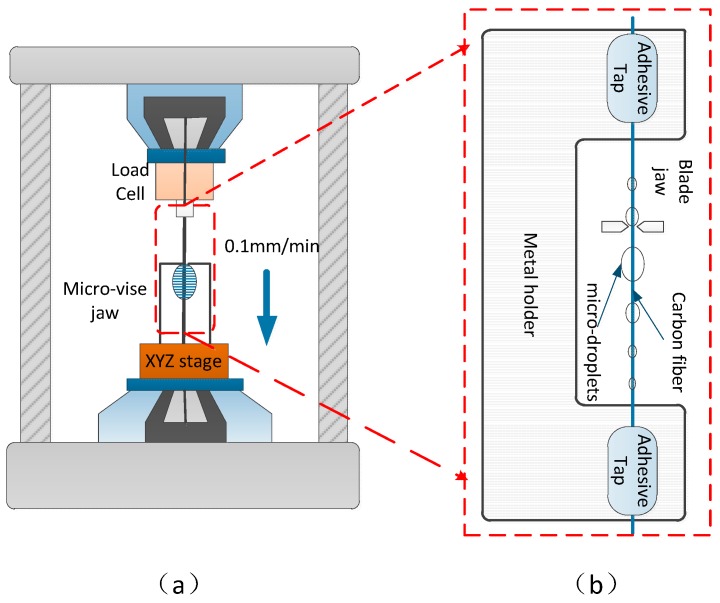
Details of microdroplet test: (**a**) Schematic of microdroplet experimental platform; (**b**) single fiber specimen for microdroplet testing.

The sample preparation diagram of the microdroplet experiment is shown in Figure 3b. We installed a 3-N load cell and an XYZ stage attached with a microvise in the tester (Figure 3a). The gap between a pair of microvise tips was maintained at about 30 μm considering the meniscus size of the droplet and the fiber diameter of 7 μm. If the gap between the two microvise heads is too small, then it may lead to frequent abnormal fractures on the single fiber. The gap is too large to effectively peel off microdroplets. The center of a single fiber was positioned at the center of the gap of the collet by incremental adjustments of the XYZ stage. After fixing the test piece, the microvise jaw was moved to load the droplet at a displacement rate of 0.1 mm/min.

## 4. Results and Discussion

### 4.1. Surface Morphology of CF

**Figure 4:**
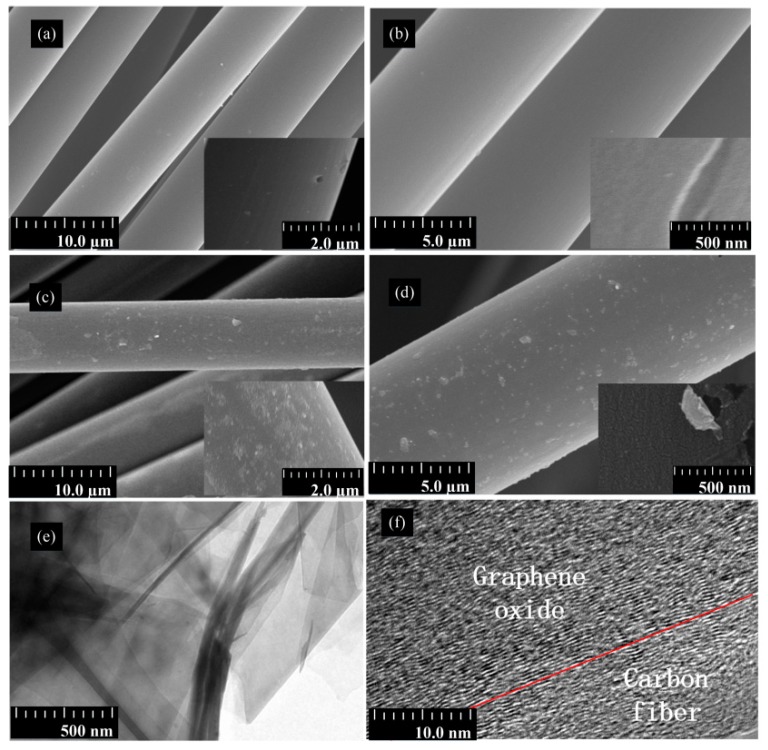
Field emission scanning electron microscopy image of CF. Panels (**a**,**b**) show the defect of CF, Panels (**c**,**d**) is images of CF–GO; panels (**e**,**f**) show the transmission electron microscope image of GO–CF.

The surface topography of the CF was characterized by SEM. SEM images of desized CF and CF–GO are shown in Figure 4. The surface of the as-received CF was defective, and some obvious micro-pores were randomly distributed along the longitudinal direction of the CF (Figure 4a,b). The morphology of GO grafted CFs changed markedly versus desized samples (Figure 4c,d). After GO grafting, the smooth surface of the CF became coarse. The surface of the CF–GO has a high GO density and uniform morphology (Figure 4c), which reveals the homogeneous distribution of GO on the surface of CF–GO. Figure 4e,f show the graphene oxide and the interface of graphene oxide on the carbon fiber surface, respectively. To clarify the existed interface properties between carbon fiber and graphene oxide, we have characterized fracture surface morphologies with SEM, as shown in Figure S1a. It is shown that two different layers exist, which implies that graphene oxide has been grafted on the carbon fiber. To illustrate the interface microstructures of these two layers, TEM characterization was further conducted. As shown in Figure S1b, graphene oxide was immersed into the graphitic layers of the CF surface. During preheating, the epoxy groups were opened as expected and reacted with –COOH and –OH groups on the surface of CF to enhance the interaction of CF and GO. Moreover, the GO on the surface of GO–CF can shrink and crack into a scale structure after heat treatment. Fortunately, this scale structure improves the interfacial properties of CF/matrix composites as well as the mechanical properties of the fibers. Upon these results, we can briefly conclude that graphene oxide is grafted onto the CF surface, which will play a positive role in repairing the CF defects.

### 4.2. Raman Characterization

**Figure 5:**
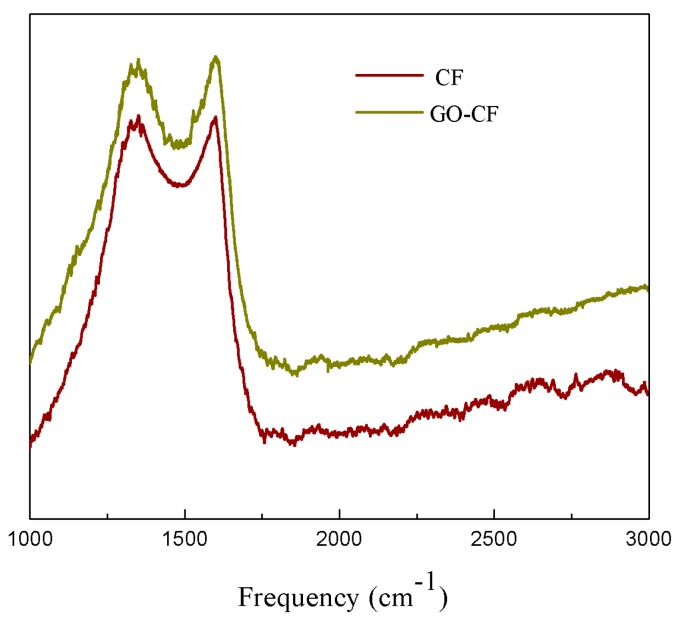
Raman spectra of CF before and after GO grafting.

Raman spectroscopy is a conventional nondestructive analysis technique for carbon-based materials, which can effectively evaluate the order and randomness of carbon-based materials [22]. The Raman spectra show D and G peaks that are characteristic of the disordered carbon and GO. The relationship between intensity and Raman shift determines the type of nanostructure present in the sample. Figure 5 shows the Raman spectra of desized CF and CF–GO in the Raman shift range of 1000–3000 cm^−1^. The peaks at around 1350 cm^−1^ and 1600 cm^−1^ are the D band from the amorphous structures of carbon and the G band from the graphitic structures of carbon, respectively [23].

In GO, the G-band shifted to higher frequency 1598.83 cm^−1^ because of oxidation of graphite. The D-band appeared at 1351.01 cm^−1^ with higher intensity. The shift in the G-band arises because of an increase in the number of sp3 carbons due to oxidation, whereas the intense D-peak is a result of imperfection created by in-plane hetero-atom oxygen-based functional groups on the graphitic basal plane. Figure 5 shows that the value of D/G (the area ratio of D to G band) in the Raman spectrum of CF–GO was also larger than that of desized CF. The larger area ratio of the D to the G band indicated that CF–GO possessed more modes of defect/disorder because the sp2 carbon network was broken into a nanoscale oxidized domain during GO fabrication [24]. In summary, GO was successfully grafted onto CF. The GO increased the surface polarity and enhanced interfacial adhesion of the fiber matrix [25].

### 4.3. Tensile Strength of the CFs

To investigate the effects of graphene on the tensile strength of a single CF, the tensile strength of desized CF and GO–CFs was characterized via a monofilament tensile test. Various defects are randomly distributed on the surface and interior of CF, and the strength of the CF is variable. Weibull statistical methods can calculate the expected tensile strength of the fiber [26]. Here, two parameters were used in the Weibull equation to analyze data from a single fiber tensile strength, namely:

$$F\left(\sigma_{i}\right)=1-\exp\left[-{\left(\frac{\sigma_{i}}{\eta }\right)}^{\beta }\right],$$

where $\sigma_{i}$ is the order ranking specimen according to size of σ, $F\left(\sigma_{i}\right)$ is the cumulative probability of single fiber failure at load $\leq \sigma_{i}$, $\sigma_{i}$ is the failure load at a certain moment i=1,2……n, β is the Weibull Shape parameter, and η is the Weibull scale parameter. Terms η and β are both constants [27]. The density function can be expressed as:

$$f\left(\sigma_{i}\right)=\left(\frac{\beta }{\eta }\right){\left(\frac{\sigma_{i}}{\eta }\right)}^{\beta -1}\exp\left[-{\left(\frac{\sigma_{i}}{\eta }\right)}^{\beta }\right].$$

The relationship between the survival probability $R\left(\sigma_{i}\right)$ and the cumulative probability $F\left(\sigma_{i}\right)$ can be obtained by:

$$R\left(\sigma_{i}\right)=1-F\left(\sigma_{i}\right)=\exp\left[-{\left(\frac{\sigma_{i}}{\eta }\right)}^{\beta }\right].$$

The formula is also simplified as follows:

$$\ln\left[-\ln\left(1-F\left(\sigma_{i}\right)\right)\right]=\beta \left(\ln\sigma_{i}-\ln\eta \right).$$

A higher value of β suggests fewer defects, a lower intensity dispersion, and better performance [28,29]. The Weibull shape parameter β is acquired via median rank estimation:

$$R\left(\sigma_{i}\right)=1-\left(i-0.3\right)/\left(n+0.4\right).$$

The term $F\left(\sigma_{i}\right)$ can be simplified via the following formula:

$$F\left(\sigma_{i}\right)=1-R\left(\sigma_{i}\right)=\left(i-0.3\right)/\left(n+0.4\right),$$

where n is the total number of single fibers tested, and i is the number of fibers broken under tensile stress $\sigma_{i}$. The expectation values ($\sigma_{0}$) of tensile strength calculated using the Weibull statistical method and the mean values ($\bar{\sigma }$) of the actual measurements are both listed in Table 1.

**Figure 6:**
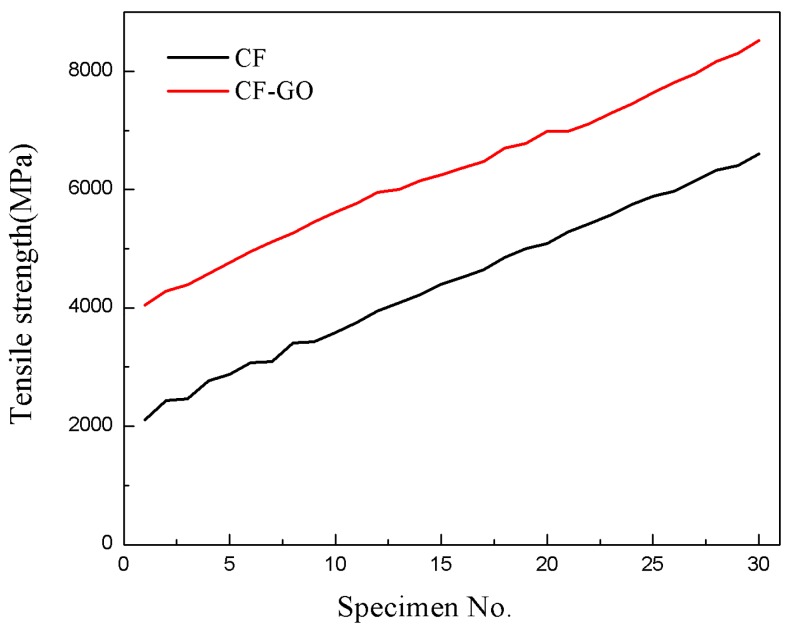
Tensile strength test curve of the CF and CF–GO.

**Figure 7:**
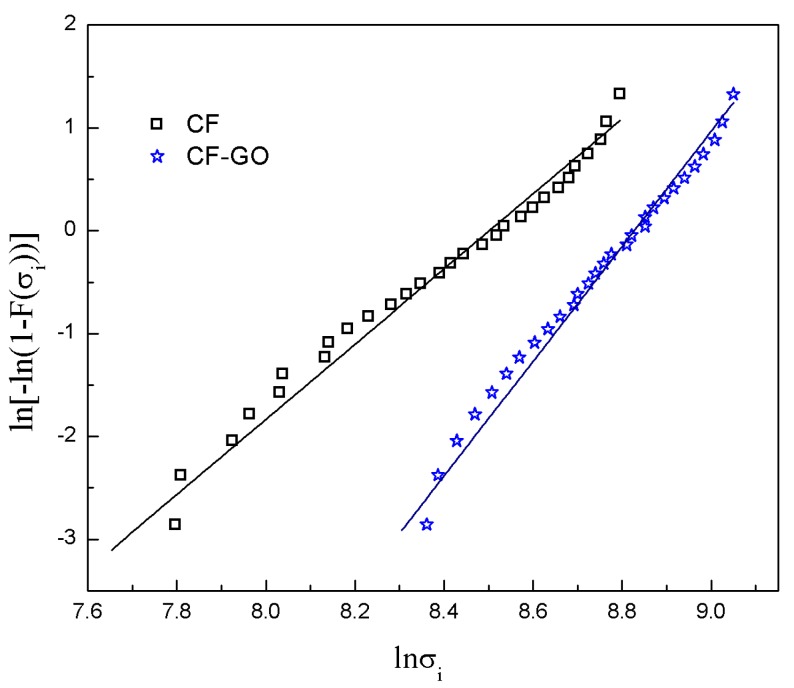
Weibull plots for tensile strength of the CF and CF–GO by monofilament.

Figure 6 shows the tensile strength of GO–CFs compared to that of CF. The tensile strength of the GO–CF increased to approximately 42% higher than that of raw CF. The Weibull fittings for the fibers before and after grafting are shown in Figure 7. The Weibull shape parameter of the GO–CF increased, i.e., it was 52% higher than that of CFs. Briefly, some large size defects on the surface of CF were cured after thermal treatment. The tensile strength of modified CF also increased significantly. The reason for the improvement is likely that the GO filled into the large defects on the surface of CF, and some active groups of the GO reacted with the surface defects to decrease the stress concentration and increase the tensile strength. Moreover, the rough surfaces formed by grafting provide extra resistance against external loading. Finally, the addition of GO sheets promoted random dispersion of particles within the regions around the fiber surface, and we can also determine that GO provides a strengthening mechanism by bridging the surface microcracks [30]. 

### 4.4. Interfacial Shear Strength of CFs

**Figure 8:**
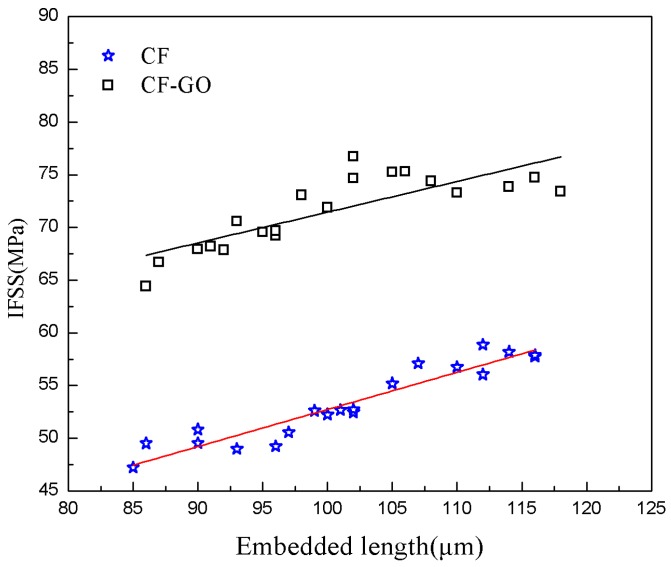
Regression approximation for the data of the interfacial shear strength (IFSS) versus embedded lengths of the microdroplet specimens.

The IFSS of CF/epoxy composites reinforced by the desized and graphene-grafted CF are shown in Figure 8. The expectation values of IFSS are listed in Table 1. The microdroplet test results show that the IFSS increased from 53.31 for the desized CF to 71.27 MPa for the CF–GO. This improves them by 33.7% versus desized CF composites.

We suggest that the IFSS improvement is partially due to the increased surface energy and the increased roughness and addition of active functional groups due to addition of GO into the interfacial region. Hydroxyl groups and carboxyl groups on the fiber surface reacted with epoxy groups and formed a chemical combination between the fiber and the GO. In brief, the chemical bond becomes stronger and cohesive failure occurs in composites of CF–GO and epoxy. The chemical interlocking plays a very important role in improving the IFSS of CF and GO.

## 5. Conclusions

Here, an efficient method for repairing CF surface defects is proposed to grow graphene sheets on the surface of CF. The thickness of the precipitated graphene is directly controlled by the thickness of the initial a-C layer and metal particle film. We then used Raman analysis to show factors affecting the formation and quality of graphene layers. The single filament tensile test proves that the use of graphene to repair the surface structural defects of CF can strengthen the axial tension of the fiber. The microdroplet experiment shows that graphene plays a positive role in the IFSS between the composite material and CF. Future work will study the physical force applied on the surface of CF substrate. 

## Figures and Tables

**Table 1:** Tensile strengths and Weibull moduli of CFs before and after grafting GO.

Fiber	d	*L* (mm)	Number	β	$\sigma_{0}\;(\mathrm{GPa})$	$\bar{\sigma }\;(\mathrm{GPa})$
Raw fiber	7(±0.23)	20	30	3.6556	4.922(±0.340)	4.349
Graphene grafted	7(±0.27)	20	30	5.5865	6.815(±0.47)	6.177

## Supplementary Materials

The following are available online at https://www.mdpi.com/1996-1944/12/8/1244/s1, Figure S1: the fracture surface of the GO-CF?
