# Supplementary material for: Amorphous Carbon-Induced Surface Defect Repair for Reinforcing the Mechanical Properties of Carbon Fiber
Source: Materials (Basel). 2019 Apr 16;12(8):1244. doi: 10.3390/ma12081244 (PMC6514943; doi:10.3390/ma12081244)
Supplement: Supplementary file 1 [file materials-12-01244-s001.pdf]

materials

Supplementary

# Amorphous Carbon-Induced Surface Defect Repair for Reinforcing the Mechanical Properties of Carbon Fiber

**Dachao Li, Hongzhong Liu *, Bangdao Chen *, Dong Niu, Biao Lei, Guoyong Ye, Weitao Jiang, Yongsheng Shi, Lei Yin and Guoquan Lai**

State Key Laboratory for Manufacturing Systems Engineering, Xi'an Jiaotong University, Xi'an 710049, China; dachaozaiwf@163.com (D.L.); dongniu@mail.xjtu.edu.cn (D.N.); biaolei@mail.xjtu.edu.cn (B.L.); guoyongye@mail.xjtu.edu.cn (G.Y.); wtjiang@mail.xjtu.edu.cn (W.J.); shiyongsheng@mail.xjtu.edu.cn (Y.S.); leiyin@mail.xjtu.edu.cn (L.Y.) wrfd92@163.com (G.L.)

* Correspondence: hzliu@mail.xjtu.edu.cn (H.L.); bdchen@mail.xjtu.edu.cn (B.C.)

Received: 14 March 2019; Accepted: 10 April 2019; Published: 16 April 2019

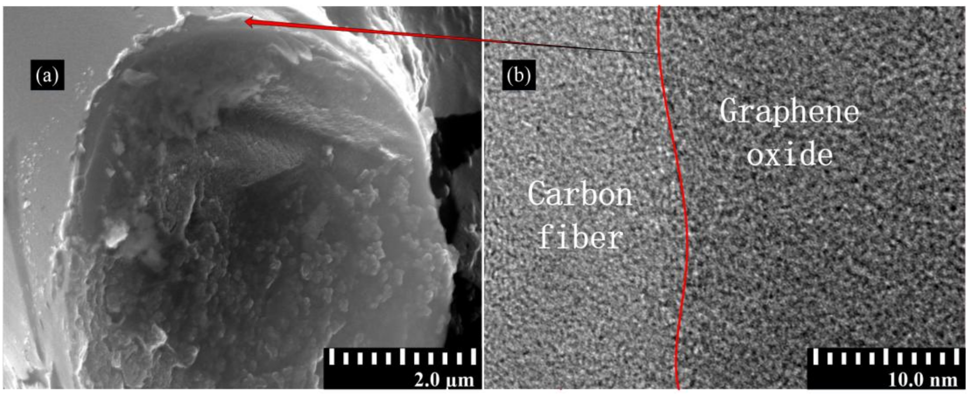


**Figure S1.** the fracture surface of the GO-CF?

To clarify the existed interface properties between carbon fiber and graphene oxide, it is of importance to demonstrate fracture surface of CF-GO after repairing. We have characterized fracture surface morphologies with SEM, as shown in Figure S1a. It is shown that apparent two different layers existed, which implied that graphene oxide has been grafted on the carbon fiber. To illustrate the interface microstructures of these two layers, TEM characterization have been further conducted. As shown in Figure S1b, graphene oxide has been immersed into the graphitic layers of the CF surface, which may be attributed to generating new cross-links between adjacent crystals during the heat treatment. Upon these results, we can briefly conclude that graphene oxide is grafted from the CF surface, which will play a positive role in repairing the CF defects.
